# Feeding Patterns of Potential West Nile Virus Vectors in South-West Spain

**DOI:** 10.1371/journal.pone.0039549

**Published:** 2012-06-22

**Authors:** Joaquín Muñoz, Santiago Ruiz, Ramón Soriguer, Miguel Alcaide, Duarte S. Viana, David Roiz, Ana Vázquez, Jordi Figuerola

**Affiliations:** 1 Estación Biológica de Doñana (CSIC), Seville, Spain; 2 Servicio de Control de Mosquitos, Diputación de Huelva, Huelva, Spain; 3 Department of Organismic and Evolutionary Biology, Harvard University, Cambridge, Massachusetts, United States of America; 4 CNM-Instituto de Salud Carlos III, Majadahonda, Spain; Global Viral Forecasting Initiative, United States of America

## Abstract

**Background:**

Mosquito feeding behaviour determines the degree of vector–host contact and may have a serious impact on the risk of West Nile virus (WNV) epidemics. Feeding behaviour also interacts with other biotic and abiotic factors that affect virus amplification and transmission.

**Methodology/Principal Findings:**

We identified the origin of blood meals in five mosquito species from three different wetlands in SW Spain. All mosquito species analysed fed with different frequencies on birds, mammals and reptiles. Both ‘mosquito species’ and ‘locality’ explained a similar amount of variance in the occurrence of avian blood meals. However, ‘season of year’ was the main factor explaining the presence of human blood meals. The differences in diet resulted in a marked spatial heterogeneity in the estimated WNV transmission risk. *Culex perexiguus*, *Cx. modestus* and *Cx. pipiens* were the main mosquito species involved in WNV enzootic circulation since they feed mainly on birds, were abundant in a number of localities and had high vector competence. *Cx. perexiguus* may also be important for WNV transmission to horses, as are *Cx. pipiens* and *Cx. theileri* in transmission to humans. Estimates of the WNV transmission risk based on mosquito diet, abundance and vector competence matched the results of previous WNV monitoring programs in the area. Our sensitivity analyses suggested that mosquito diet, followed by mosquito abundance and vector competence, are all relevant factors in understanding virus amplification and transmission risk in the studied wild ecosystems. At some of the studied localities, the risk of enzootic circulation of WNV was relatively high, even if the risk of transmission to humans and horses was less.

**Conclusions/Significance:**

Our results describe for first time the role of five WNV candidate vectors in SW Spain. Interspecific and local differences in mosquito diet composition has an important effect on the potential transmission risk of WNV to birds, horses and humans.

## Introduction

West Nile virus (WNV) is a zoonotic mosquito-transmitted arbovirus whose enzootic cycle is maintained by birds and mosquitoes [1]. Virus amplification and maintained circulation require successive feeding events in viraemic birds but transmission to humans and/or horses (i.e. dead-end hosts) can cause disease outbreaks [2]. It is well known that virus amplification is affected by the characteristics of the mosquito species (i.e. its vector competence [3]), vertebrate host characteristics (i.e. host competence [4]) and abiotic factors (i.e. temperature and rainfall [5]). One potentially important variable in the understanding of virus amplification and transmission is the degree of vertebrate-mosquito contact or, in other words, the degree of interaction between mosquitoes and their hosts, since not all vertebrates are competent hosts for this virus or are susceptible to this disease [4].

The use of serological and molecular methods has greatly improved our capacity for describing feeding patterns of haematophagous arthropods in the wild [6], [7]. These approaches have allowed us to detect blood from a large variety of vertebrates in mosquitoes and to identify important differences in diet between seasons and/or localities [8], [9]. Previous studies have focused on mosquito feeding behaviour in rural, urban and peri-urban zones (see [10]-[13]). Nevertheless, natural ecosystems that contain diverse and abundant mosquito communities, as well as a wide range of vertebrate hosts, have to date been poorly studied (e.g. [14]). Aside from mosquito feeding preferences, vector competence and mosquito abundance are also significant parameters in the amplification of WNV [15] and could be related to virus epidemics.

Currently, most of our knowledge of WNV ecology and dynamics comes from North America as a result of the introduction of this arbovirus in 1999. Since its rapid expansion throughout this continent, WNV has caused important human and wildlife health problems [16]. Nevertheless, in Europe the patterns of WNV circulation are still unclear. WNV outbreaks occur nearly every year but in different and often widely separated regions. A number of studies have proposed that this pattern is the result of infected migratory birds arriving from Africa and seeding the virus in different areas [17]. However, other results suggest that the virus remains endemic all year in some areas of Europe, but only becomes epidemic in horses or humans under certain conditions [18]. Seroconversions in wild animals and virus isolation in mosquitoes suggest that WNV has been circulating in Spain regularly at least since 2003 [19]-[21], during which time cases of illness have been reported sporadically in birds, horses and humans [22] (ProMED-mail, Archive Numbers: 20101119.4203 and 20100925.3478). Despite the potential importance for understanding patterns of WNV (and flavivirus in general) amplification and transmission, very little is known about mosquito feeding patterns in Spain and in Europe in general [23]-[27].

In the present study, first of all we use molecular methods to describe feeding patterns in five mosquito species captured in three different wetlands in SW Spain (Doñana, Odiel and La Janda) from 2007 to 2009. We then quantify the relative influence of inter-specific, spatial and temporal variation on mosquito feeding patterns. Finally, we estimate the transmission risk of WNV in birds, horses and humans for different mosquito species and localities, and evaluate the sensitivity of these estimates to the natural range of variation in three important parameters for the transmission of WNV.

## Methods

### Study Area and Mosquito Samples

Our study area consists of a ‘multi-ecosystem’ landscape (e.g. forests, dunes, beaches, scrubland, marshland and rural settlements). The Doñana Natural Space occupies over 1,060 km^2^ [28] and receives around 350,000 visitors a year (data from last three years), with peaks during spring and summer (mainly in March–May and July–August). This area is considered one of the most important and outstanding protected spaces in Europe and is a major wintering and stopover area for migratory birds. Previous studies performed in this area have documented the circulation of WNV between resident birds [19], [29] and horses [30]. The presence of WNV, Usutu virus (USUV) and other flaviviruses in various mosquito species were detected during the sampling period for the present study [20], [21], although no case of WNV-related illness was reported. The estuary of the Odiel river (Huelva province) covers 10,000 ha of tidal marshland in close proximity to a coastal area frequented by large numbers of tourists in summer. La Janda (Cádiz province) consists of 3,000 ha of rice paddies on the site of a drained lake.

**Figure 1:**
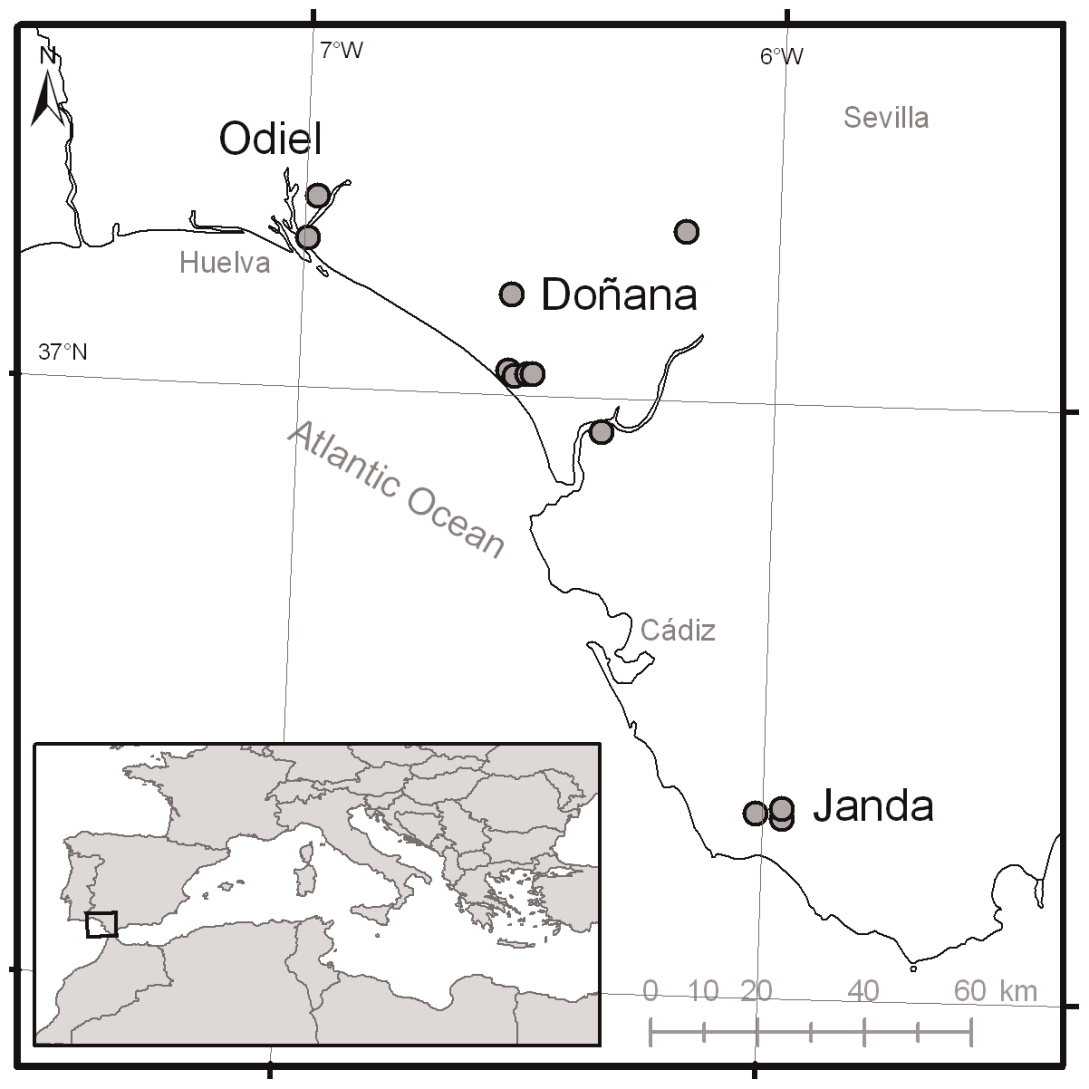
Sampling sites in the study area in SW Spain (the three studied wetlands were: Odiel, La Janda and Doñana – see text for details).

CDC light-traps baited with CO_2_ were placed at 12 sampling sites (Fig. 1) and operated for 24 hours every 1–2 weeks between March and November for three years (2007–2009). Trapping of mosquitoes was done with all the necessary permits from the regional Department of the Environment (Consejería de Medio Ambiente, Junta de Andalucía). Samples were preserved in dry ice and then transported to the laboratory for identification of species, gender and feeding status. Specimens were separated using Petri plates with white filter paper on a chill table and identified following the appropriate identification keys [31], [32] using a stereomicroscope. *Culex* mosquitoes belonging to the *Univittatus* complex were named as *Culex perexiguus* based on male genitalia following Harbach [33]. Individual engorged females, identified visually by abdominal blood contents, were stored at −20°C for subsequent blood meal analyses. The number of mosquito females captured per sampling site ranged from 2,490 to 61,984, while the number of females captured per species ranged from 12,846 (*Cx. pipiens*) to 99,191 (*Cx. theileri*), see Table 1.

**Table 1:** Origin of the blood meals identified in five different mosquito species during 2007–2009.

	Hosts					
Mosquito species	Avian	Mammal	Reptilian	Horse	Human	Total per species (specimens sampled)
*Culex modestus*	41	5	2	0	0	48 (16,175)
*Culex perexiguus*	49	14	0	3	0	63 (28,226)
*Culex pipiens*	69	11	1	0	4	81 (12,846)
*Culex theileri*	61	407	0	49	7	468 (99,191)
*Ochlerotatus caspius*	47	204	0	22	3	251 (56,659)

The number of blood meals on horses and humans are also included in the mammal data.

### Molecular Identification of Blood Meals

Individual mosquito abdomens were separated from the rest of the insects’ bodies and put into individual PCR tubes using two disposable pipette tips per specimen. DNA was isolated from the abdominal contents using the HotSHOT protocol as described in [6]. DNA extracts from the mosquito blood meals were used for a first standard Polymerase Chain Reaction (PCR) assay, whose PCR products were subsequently used for a nested PCR to amplify a fragment of vertebrate Cytochrome *c* Oxidase Subunit I (COI) mitochondrial gene. For both PCR assays we used previously described primers and thermal-cycling conditions [6] with a PTC-100 Programmable Thermal Controller (MJ Research). Amplicons of the appropriate size, as determined by agarose gel electrophoresis were purified using the commercial ExoSAP-IT reagent (GE Healthcare Life Sciences). Sequencing reactions were performed according to BigDye 1.1 technology (Applied Biosystems) using BCV-RV2 primer. Labelled DNA fragments were resolved through an ABI 3130xl automated sequencer (Applied Biosystems). Sequences were analysed and annotated using the software Sequencher™ v.4.5 (Gene Codes Corp., © 1991–2005) and identified by comparison with the GenBank DNA sequence database (National Center for Biotechnology Information, 2008) and the BOLD Systems platform (http://www.boldsystems.org/views/login.php) to assign unknown COI sequences to particular species. Positive identification and host species assignment were made when exact or nearly exact matches (>98%) were obtained and differentiated from the nearest most similar species by around 2%.

### Statistical Analyses

To explain the presence of bird, human, horse and mammal blood in mosquitoes, we fitted Generalized Linear Mixed Models (GLMM) with binomial error distribution using ‘mosquito species’, ‘wetland’, ‘locality’ nested on ‘wetland’, ‘season’ and ‘year’ as random factors. Variance components were used to estimate the relative (%) contribution of each of these random factors to diet variation and the statistical significance determined by Likelihood Ratio tests. Four different models were built to analyse the factors related to the presence of bird, human, horse and mammal blood (including horse and human blood meals). For example, for the analyses of avian blood meals the dependent variable was scored as 1 when avian blood was detected in a female mosquito and 0 if only blood from other vertebrate groups was detected. Models were fitted with the Procedure GLIMMIX implemented in SAS 9.2. (SAS-Institute, Cary, NC, USA) using Laplace approximation, since it is considered to be more adequate than Pseudo-likelihood when fitting mixed models with binary response variables [34].

Rarefaction-based estimates on host richness were computed using the software Analytic Rarefaction v.2.0 (http://www.huntmountainsoftware.com/html/rarefaction.html) for each mosquito species.

### Estimation of WNV Transmission Risk

The relative risk that a species of mosquito might infect humans, horses or birds with WNV was estimated using Kilpatrick’s formula [15], which we modified by not including the Minimum Infection Rate (MIR) values for each mosquito due to the lack of information on WNV infection rates for most of the species and localities analysed in our study. In our formula (see below) we used the proportion of avian blood meals as an indicator of the exposure of each mosquito species to WNV, since only in some bird species viraemia is high enough to infect the mosquitoes that feed on them. Unfortunately, host competence values for avian species in Europe do not exist for as many species as in North America and so for the moment it is not possible to conduct more detailed analyses that divide avian species into competent and non-competent hosts. Although high viraemias have been reported in several mammal species [35], none of these species occur in our study areas. Our risk transmission estimation is a relative measure of the number of WNV-infectious bites that a host species or group (e.g. humans, horses or birds) receives from a mosquito species in relation to mosquito abundance, its vector competence and its feeding pattern. We applied the following formulas for each mosquito species and locality:
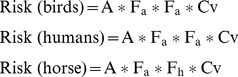
where A is the abundance of mosquitoes estimated as the mean number of females captured per trap/night, F_a_ is the fraction of blood meals taken from avian hosts, F_H_ the fraction of blood meals taken from humans, and F_h_ the fraction of blood meals taken from horses. Cv is a measurement of the vector competence estimated as the proportion of bites from infected mosquitoes that transmit the virus. Given that no data on vector competence from Spain are available, we used previously reported data from France and South Africa [3], [36]. Our results are given as an index representing the potential total risk to humans, horses and birds for each mosquito species (see [15]).

To determine the importance of natural variation in each parameter for potential risk of WNV transmission, we performed a sensitivity analysis using the R package [37], [38]. We estimated the Sobol Index values according to the method described in [39], [40] for each parameter. These values represent the amount of variation in transmission risk that can be attributed to each parameter, both due to its pure effect (i.e. first-order index) and to interactions with other parameters (i.e. total index). In order to increase the coverage of the sampled distributions (i.e. uniform) and all possible combinations of the values from all parameters, a Latin hypercube sampling (LHS) technique was applied [41]. Briefly, two 10,000 (number of samples) by six (parameters) pseudo-random matrices were created to allow parameters to vary uniformly within their real ranges at the sampling sites. The range of variation for each parameter used were: blood meal component (F_a_ ranging between 0 and 1; F_H_ ranging between 0 and 0.2; and F_h_ ranging between 0 and 1), mosquito abundance (A, ranging between 0.21 and 326), and virus competence (Cv, ranging between 0.1 and 0.9). Each combination corresponding to the blood meal fractions was forced to add up to one, but no restrictions were placed on the values of the other two parameters.

## Results

### Mosquito and Host Identification

We identified ten mosquito species from a total of 221,819 samples, of which only five (*Culex modestus*, *Cx. perexiguus*, *Cx. pipiens*, *Cx. theileri* and *Ochlerotatus caspius*) provided enough blood meals to perform statistical analyses. All of these five species are considered potential WNV vectors [3], [31]. A total of 911 blood meal DNA extracts were amplified via PCR, and then sequenced and identified to species level (see Table 1).

**Figure 2:**
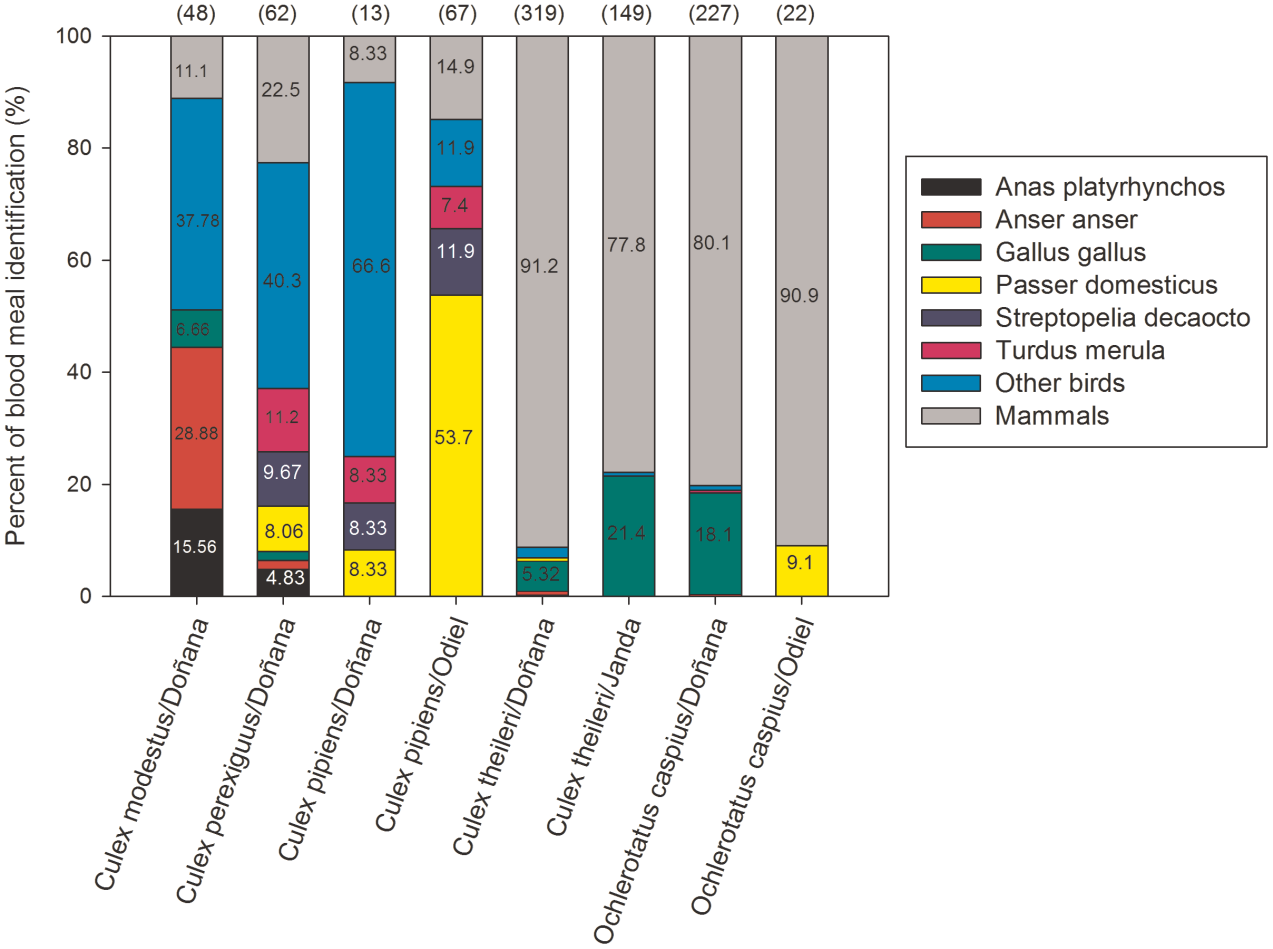
Origin of blood meals of the five mosquito species analysed in the three different wetlands in SW Spain. Only the main bird and mammal species are shown. Numbers above bars indicate the number of bloodmeals per mosquito species obtained for each sampled wetland. Percentage of blood meals for each vertebrate species/group are give as numbers inside the bars when higher than 5%. Only species/wetlands with more than 10 bloodmeals were included in the figure.

**Figure 3:**
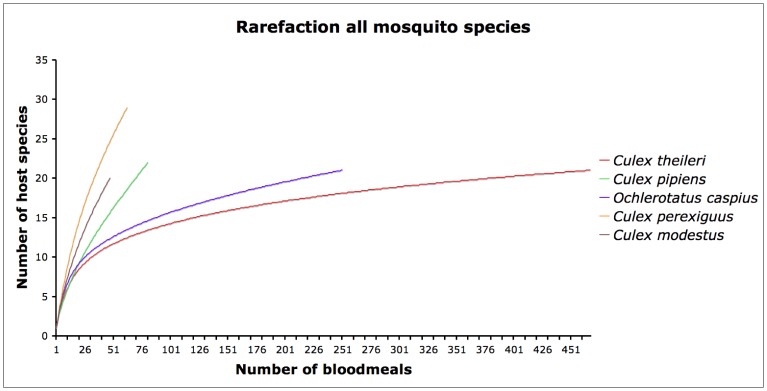
Rarefaction curves of the number of host species detected in relation to the number of female mosquito blood meals analysed.

We identified 61 vertebrate host species, which included 42 birds, 17 mammals and two reptiles (see Table 1 and Fig. 2); only *Culex modestus* and *Cx. pipiens* were found to feed on reptiles. *Culex perexiguus* fed on 29 host species (22 birds and seven mammals). The number of host species identified for the other four mosquito species ranged between 20 and 22. Rarefaction curves (Fig. 3) grew rapidly at first, although in three *Culex* species (*Cx. modestus*, *Cx. perexiguus* and *Cx. pipiens*) the number of host species detected did not reach a plateau.

### Factors Driving Mosquito Feeding Patterns

Patterns of variation in the prevalence of avian, mammal and horse blood meals were similar (see Table 2). Only ‘mosquito species’ (around 49–65% of explained variance) and ‘locality’ (around 30–50% of explained variance) were significantly associated to blood meal origin. ‘Season’ and ‘study year’ also explained a significant amount of variance in the presence of horse blood meals, but none in the case of avian and mammal blood meals. However, a very different pattern was found in the case of human blood meals, which were concentrated in summer, the only season that was significantly related to human blood presence (Table 2). ‘Mosquito species’, ‘locality’ and ‘year’ were unrelated to human blood presence. ‘Wetland’ did not explain a significant amount of variance in mosquito feeding on any of the vertebrate groups or species considered.

**Table 2:** Explained variance and statistical significance of mosquito species (Species), Wetland, Locality, Season and Year in relation to the presence of bird, mammal, human and horse blood in female mosquitoes.

	Host identified from bloodmeals											
	Avian			Mammal			Human			Horse		
Explanatory variable	% variance	χ^2^	*p*-value	% variance	χ^2^	*p*-value	% variance	χ^2^	*p*-value	% variance	χ^2^	*p*-value
Species	49.16	80.50	<0.0001	52.29	88.31	<0.0001	0.00	0.00	1.00	64.80	19.90	<0.0001
Wetland complex	0.00	0.00	1.00	0.00	0.00	1.00	13.04	1.01	0.31	0.00	0.00	1.00
Locality	49.85	191.01	<0.0001	46.59	188.00	<0.0001	0.00	0.00	1.00	30.16	28.03	<0.0001
Season	0.99	0.99	0.32	1.12	1.38	0.24	66.33	4.23	0.04	3.52	5.64	0.02
Year	0.00	0.00	1.00	0.00	0.00	1.00	20.63	1.48	0.22	1.52	3.59	0.05

### Potential Transmission Risk to Avian Species, Horses and Humans

**Figure 4:**
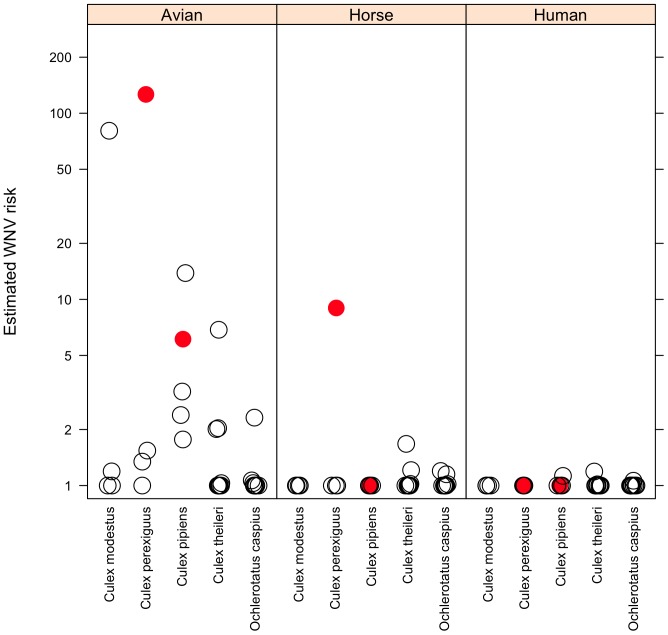
WNV transmission risks for each different mosquito species estimated from the 12 sampling sites for birds, horses and humans. For localities with the same estimates dots have been slightly displaced horizontally to avoid complete symbol overlapping. Solid circles indicate those mosquito species and sampling sites in which WNV has been reported in mosquitoes (Vázquez et al. [20], [21]). Estimates are reported for 4 localities for *Cx. modestus*, 4 for *Cx. perexiguus*, 5 for *Cx. pipiens*, 10 for *Cx. theileri*, and 10 for *Oc. caspius*. One was added to all estimates to allow plotting on a log10 scale.

*Culex perexiguus* had the highest potential for enzootic virus transmission, with a mean bird-to-bird estimated transmission risk of 31.52±62.4 (S.D.), followed by *Cx. modestus* (19.9±39.7) and *Cx. pipiens* (4.6±4.9). *Culex perexiguus* also had the highest transmission risk to horses (1.99±3.4), followed by *Cx. theileri* (0.1±0.2), although this risk was very low in comparison to birds. The potential transmission risk to humans was low for *Cx. pipiens* (0.025±0.05), *Cx. theileri* (0.02±0.05) and *Oc. caspius* (0.006±0.01) (see Fig. 4).

**Figure 5:**
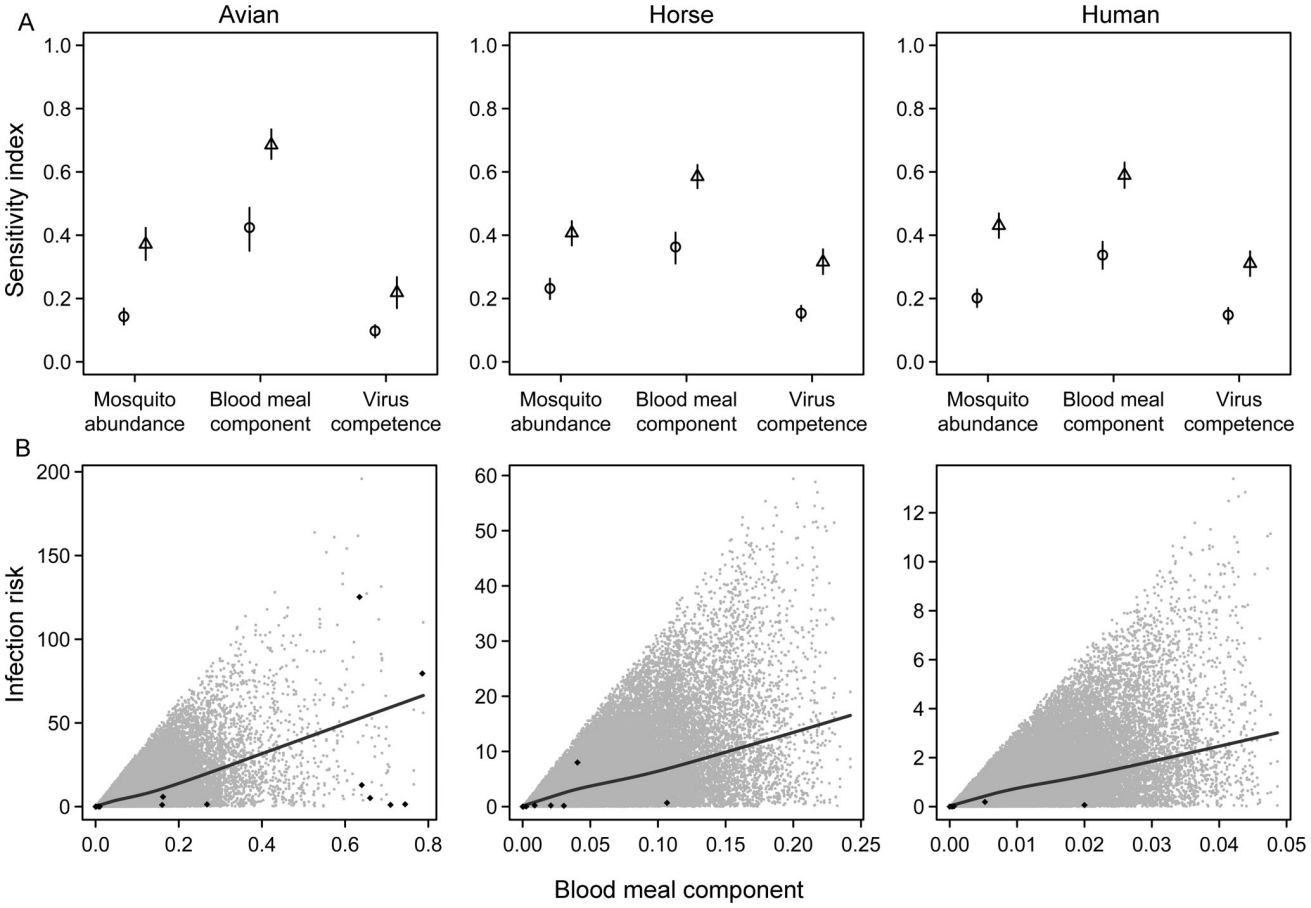
Results of sensitivity analyses of estimated WNV transmission risk: **A**. First order (circles) and total (triangles) sensitivity Sobol Index values for birds, humans and horses are given. Bars denote 95% confidence intervals. **B**. Relationship between the blood meal component and the infection risk estimated in the sensitivity analysis; i.e. Fig. A (lower panel; grey dots). The blood meal component is calculated as the product of mosquito blood meals on birds and mosquito blood meals on the group in question (birds, humans or horses). The black line is a lowess (locally weighted scatterplot smoothing) smoother that helps visualize the positive tendency between the parameter and the risk of infection; black diamonds correspond to risk values at the sampling locations.

The sensitivity analysis suggested that ‘blood meal origin’ makes a greater contribution to transmission risk than either ‘mosquito abundance’ or ‘vector competence’ (Fig. 5A). The estimated values of enzootic transmission risk are large in some of our study areas when compared with theoretical values (Fig. 5B), but tend towards a lower range of possible values for potential transmission risk in humans and horses.

## Discussion

Our results showed that the studied mosquito species fed on a wide range of vertebrates (e.g. birds, mammals and reptiles) and thus all of them can be considered as generalists (see Fig. 2), as has been reported in other regions of the world [31], [42]. However, important inter-specfic differences in the occurrence of avian and mammal blood meals were detected: *Culex modestus*, *Cx. perexiguus* and *Cx. pipiens* (i.e. the main WNV vectors, see below) feed mainly on birds (78–85% of blood meals), while *Cx. theileri* and *Oc. caspius* feed mainly on mammals (81–87%) (Fig. 2). Interestingly, the rarefaction curves of the former three *Culex* species had the steepest gradients since they fed on a large number of hosts, which translates into a potentially more complex WNV enzootic cycle involving a larger number of avian host species. The techniques used to trap the mosquitoes may also potentially affect the characteristics of the sampled fraction of the population. CDC traps baited with CO_2_ capture preferentially host-seeking females or females that have not been able to successfully complete a blood meal (i.e. because they have fed on hosts with strong anti-mosquito behaviour) [43]. However, it is important to note that we found no differences in the vertebrate origin of blood meals between female mosquitoes captured with CDC light traps lured with CO_2_ and those captured in resting boxes (BG traps without any attractant) [44]. Additionally, this potential bias may affect the absolute values of the parameters (i.e. the proportion of mammal blood meals) but is not likely to affect the conclusions of variance decomposition analyses or the relative estimates of transmission risk.

### Variance Decomposition Analyses

Our analyses indicate that the major factors involved in the feeding behaviour of these arthropods are the species and the sampling site where adult females are captured. Recently, Chaves et al. [10] concluded that mosquito feeding patterns were unrelated to vertebrate class (i.e. there were no inter-specific differences in preferences for mammals or birds). However, our results suggest that these authors’ conclusions are the product of an artefact caused by having ignored interaction strengths (i.e. the feeding frequency in each clade). Our results, nevertheless, highlight the need to include interaction strength (i.e. frequency) and the serious impact of differences in vertebrate-mosquito contact in the amplification and transmission risk potential of pathogens. Our rarefaction analyses indicate that larger sampling sizes than those currently used in most mosquito feeding/behaviour studies are also essential if accurate descriptions of the vertebrate species present in mosquito diets are to be obtained, above all if we aim to infer anything from the absence of a particular species from mosquito diet. Additionally, our variance decomposition analyses indicate that, in terms of the presence of vertebrate classes in mosquito diets (birds or mammals), large spatial variation exists, although ‘mosquito species’ does still explain a large amount of variance.

In addition to ‘mosquito species’, our results showed that ‘locality’ (i.e. spatial heterogeneity) is a factor that affects the origin of blood meals, although this effect occurred at the level of trap location rather than at the ‘wetland-complex’ level. It is important to note that *Culex* females do not fly long distances [31] but feed near the area of capture; consequently, vertebrate availability at such local scales will determine the origin of their blood meals. Hamer et al. [45] observed that the composition of vertebrate communities determines vector feeding patterns. Nevertheless, the nature of the relationship between vertebrate abundance and mosquito feeding is still poorly understood [10], [42], [45], [46]; for example, mosquitoes do not necessarily feed on the most abundant prey species [45], [47]. Vertebrate species may differ widely in their host competence (i.e. the sum of the probability that an infected host will transmit virus to a biting mosquito on each of the seven days following infection [4], [48]) and important differences may exist even between different species of birds [4]. Consequently, different vertebrate communities may vary dramatically in their capacity for pathogen amplification, circulation and transmission to particular host species. Unfortunately, not enough information on the host competence of European avian species exists [49] to be able to refine our analyses by dividing avian species into host-competent and non-competent groups. Thus, our results should be taken as a generalized approach in the absence of further detailed information.

In our study, the frequency of feeding on humans was only affected by the season. The low number of human blood meals reported in our study is related to the fact that we worked in natural areas with very low anthropic presence. Although the small sample size may have reduced our capacity to detect other effects (i.e. mosquito species) as statistically significant, the amount of variance explained (Table 2) does support the importance of seasonal differences in the occurrence of human blood meals. Seasonal differences in the frequency of human blood meals have been associated with the occurrence of WNV epidemics in Colorado and California, U.S.A. [50], [51], where in an urban environment the increase in feeding on humans was linked to a decrease in the abundance of the preferred avian hosts [8]. In our case, in addition to potential changes in the abundance of other preferred vertebrates, the increased presence of human blood meals during the summer season could be a reflection of the increased number of visits by humans that make them more accessible to mosquitoes. Human behaviour might have an impact on the transmission of some vector-borne virus in urban zones [52] and the same could be applicable to WNV in non-urban areas.

### Mosquito Species Relevant for WNV Transmission in SW Spain

The greatest potential risk of WNV transmission in the studied wetlands in SW Spain occurs between birds (i.e. enzootic cycle). It is fifty times less between birds and horses and two hundred times less between birds and humans (Figs. 4 and 5), although risk values were found to be very heterogeneous between localities. In fact, the WNV antibody prevalences found in Doñana for birds (10.4% [29]), horses (5.3% [30]) and humans (0.5% [53]) follow the same patterns of variation as our estimated transmission risks. However, these differences are also affected by the different age structure of the populations of these organisms and by the different duration of detectable antibodies in the bloodstream after infection by WNV.

In our study area, our results for *Cx. perexiguus* show a high potential risk of WNV transmission from bird to bird and from bird to horse, this mosquito species being the primary candidate for enzootic (bird-to-bird) transmission and for epizootic transmission to horses. *Culex pipiens* is also an enzootic vector, but has only a low risk for epizootic transmission to humans. There was also a potentially high risk of transmission for *Cx. modestus* from bird to bird; despite being a secondary enzootic vector, this species was not implicated in epizootic cycles, unlike in the Camargue in France [24], [54]. Finally, *Cx. theileri* and *Oc. caspius* can be considered secondary epizootic vectors. Interestingly, the *Cx. perexiguus* and *Cx. pipiens* positive pools for WNV detected in Spain [20], [21] were collected in the two combinations of ‘locality-mosquitoes’ ranked among the highest transmission risk values (black dots in Fig. 4). This suggests that patterns of variation in estimated WNV transmission risk may be related to real WNV transmission patterns; nevertheless, a larger sample size (i.e. number of localities with and without WNV mosquito positive pools) is needed if we are to test this relationship statistically. The rapid accumulation of detailed studies regarding the origin of blood meals should allow us to investigate in the future how these parameters differ whenever and wherever epidemics among horses and humans occur.

### Sensitivity Analyses for WNV Transmission Risk

Our sensitivity analysis ranked the blood feeding pattern (diet) as the main factor related to virus transmission, followed by mosquito abundance and vector competence. This conclusion reflects the great variability in diet composition reported in natural mosquito populations and is not an artefact caused by including two blood meal related variables as the components of diet in the formula. Note that diet is analysed in the sensitivity analyses as a simple parameter and that analyses of sensitivity conducted using the same levels of variation for the three parameters analysed (diet, mosquito abundance and vector competence) would assign the same importance to each factor. We believe that it is important to emphasize the fact that the importance of vector competence may have been underestimated in these analyses because vector competence can vary geographically [55] (but also with temperature and virus characteristics), and we have used the same values (estimated from mosquitoes Camargue in France for 3 species and from South Africa for other two species) for all the localities. However, to the best of our knowledge, no information of the potential variability in vector competence at the spatial scale analysed in our study (150 km) exists. Thus, our analyses suggest that changes in the relative frequency of blood meals in certain vertebrate groups might lead to changes in the transmission risk for potential host vertebrate species. This result reinforces the idea that broad-based surveys on host communities are needed if we are to accurately estimate transmission risks.

One lesson learnt from WNV outbreaks is that a particular outbreak is never the same as the previous one, and that the mosquito species involved in the new outbreak may differ [54]. Not surprisingly, the estimated risk of transmission for a single species varied strongly between localities (Fig. 4), thereby illustrating the potential for spatial heterogeneity in the relevance of different mosquito species for WNV circulation. Recently, Simpson et al. [51] concluded that seasonal changes in blood meal origin in *Cx. pipiens* was the more influential parameter explaining the intensity and timing of WNV transmission in different localties of North America. In our area, *Cx. perexiguus* (the European species in the *Univittatus* complex [33]), morphologically similar to *Cx. univitattus* (a major WNV vector in Africa and in the Middle East [16], [56]), is the primary enzootic vector and a potential epizootic vector for horses. These two species have been recorded as the principal vectors in several WNV outbreaks in regions such as Egypt [57], South Africa [36], [58], Kenya [59], Israel [60]; and Portugal [61]. Therefore, we should not underestimate the role of *Cx. perexiguus* as an enzootic and epizootic vector of WNV in SW Spain and Europe.

In conclusion, our study represents the first quantification of the relative importance of mosquito species, location and season in explaining differences in blood meal origin in mosquitoes from a natural wetland ecosystem and the consequences that this finding may have for WNV dynamics in Europe. The presence of human blood meals was only related to season, while the presence of avian or mammal blood meals depends on mosquito species and locality. Estimates of transmission risks had important spatial and inters-pecific differences and closely match what little data is available on WNV detection in the area. Diet composition had a stronger impact than either mosquito abundance or vector competence on estimated transmission risks due to high spatial and inter-specific differences in diet composition. Our findings provide invaluable information regarding vector surveillance for WNV activity and highlight the potential risk of WNV transmission to horses and between birds by a poorly known *Culex* species, *Cx. perexiguus*.
